# Risk Factors and Predictors of 1-Year Mortality in 262 Vancouver Type C Periprosthetic Femoral Fractures: Insights from the PIPPAS Prospective Multicenter Observational Study

**DOI:** 10.3390/jcm14175986

**Published:** 2025-08-25

**Authors:** Héctor J. Aguado, on behalf of the PIPPAS Study Group

**Affiliations:** Hospital Clínico Universitario de Valladolid, 47003 Valladolid, Spain

**Keywords:** periprosthetic fractures, Vancouver C, orthogeriatrics, frailty, femoral fracture, total hip arthroplasty, complication, mortality, risk factors

## Abstract

**Background/Objectives**: Vancouver type C periprosthetic femoral fractures (VC-PFFs) predominantly affect frail elderly patients and are associated with high mortality, yet limited evidence exists regarding prognostic factors. The PIPPAS study (Peri-Implant and PeriProsthetic Survival Analysis) sub-analysis aimed to investigate the risk factors for one-year mortality following VC-PFF and identify predictors of medical and surgical complications. **Methods**: This prospective, multicenter, observational case series was conducted across 59 hospitals in Spain and involved 262 VC-PFF patients between January 2021 and April 2023 with a minimum 1-year follow-up. Demographic, clinical, management, and surgical and outcome data were collected. Logistic regression models were used to identify predictors of one-year mortality and complications. **Results**: One-year mortality was 30.1%. VC-PFF patients were elderly (median age 85 years, IQR (12.75)), female (77.1%) and frail: median clinical frailty scale 5, IQR (2), mild cognitive impairment (median Pfeiffer score 3, IQR (5)), and multiple comorbidities (median age-adjusted Charlson comorbidity index (a-CCI) 6, IQR (2)). Surgery was performed in 94.7% of cases, primarily with plate osteosynthesis (62.3%) or intramedullary nailing (29.1%). Male sex, higher age, frailty, cognitive impairment, ASA score, and a-CCI were significantly associated with increased mortality. Protective factors included higher hemoglobin levels, surgical treatment, and early postoperative ambulation. No significant difference in mortality was observed between fixation techniques. **Conclusions**: One-year mortality in VC-PFF patients is high. These findings underscore the need for individualized treatment plans and reinforce the role of early co-management and clinical optimization.

## 1. Introduction

Periprosthetic femoral fractures (PFFs) after hip arthroplasty are a severe complication and represent a growing challenge in orthogeriatric trauma care [1,2,3,4,5,6,7]. Their incidence is rising worldwide [8], particularly as the ageing population expands and the increase in the number of hip arthroplasties. These low-energy fractures frequently affect older adults with complex comorbidity profiles, cognitive impairment, and varying degrees of frailty [9,10]; these factors complicate both surgical decision-making and postoperative recovery. PFFs are associated with severe medical complications, prolonged hospital stays, and delayed recovery [5,6,7,10]. The PFF population shows mortality rates approaching 30% at one-year post-injury, equal or higher than those observed in the broader proximal femur fracture population, aligning them with outcomes observed in hip fracture populations [4,10,11,12,13,14,15,16,17,18].

According to the Unified Classification System (UCS), Vancouver type C periprosthetic femoral fractures (VC-PFFs) are located distal to the femoral stem, typically within the diaphyseal region of the femur, and are generally characterized by an intact prosthetic implant [19]. Unlike type B fractures that may require complex revision surgery, type C fractures are commonly treated with internal fixation methods, such as locked plating or retrograde intramedullary nailing [20,21,22,23,24,25]. Moreover, the impact of patient-related variables such as frailty, cognitive status, hemoglobin levels, walking ability, and residential setting on postoperative survival remains poorly understood. Additionally, there is limited consensus in the literature regarding the optimal surgical approach [26]. Previous studies on this topic are retrospective, based on small single-hospital cohorts, cover extended time periods, and primarily focus on broader groups of PFFs without isolating type C fractures [1,23,24,25,27,28], resulting in a knowledge gap concerning prognostic indicators and treatment outcomes specific to this subgroup.

This study addresses that gap by evaluating risk factors for one-year mortality and the role of medical and surgical complications in shaping patient outcomes in a large, prospectively collected, multicenter cohort of patients with VC-PFFs. The aim is to enhance clinical decision-making by identifying modifiable risk factors and informing tailored management strategies for this vulnerable patient population.

## 2. Materials and Methods

The PIPPAS study (Peri-Implant and PeriProsthetic fractures Analysis for Survival) is a collaborative, multicenter, prospective, observational case series study (level IV evidence) evaluating periprosthetic and peri-implant fractures in fifty-six Spanish hospitals and one in Argentina, collectively representing 37.5% of the national population coverage (approximately 17.8 million individuals) [10]. VC-PFF management was the standard of care at each participating site, as determined by the attending surgeon. This cohort sub-study included patients aged 18 years or older who presented with a VC-PFF between January 2021 and April 2023, and had available 1-year follow-up clinical data. We excluded patients managed non-operatively, patients with intraoperative or pathologic fractures, patients with fractures between a hip stem and any other distal implant, and those with pregnancy. Written consent for participation in the study was obtained from all participants or their legal representatives.

Prospective data collection included patient demographics, management, and outcomes based on the Fragility Fracture Network’s Minimum Common Dataset for hip fracture audits, but adapted to the specific nature of VC-PFFs (Fragility fracture network. Minimum Common Dataset. https://fragilityfracturenetwork.org/hip-fracture-audit/, accessed on 14 November 2020) Cognitive status was assessed with the Pfeiffer Short Portable Mental Status Questionnaire (SPMSQ) [29]. Experienced surgeons were those who have performed over 20 minimally invasive fixations or arthroplasty revisions in the last 12 months. Fracture healing was defined as the presence of at least three cortical callus bridges on radiographic examination and pain-free full weight bearing. A comprehensive list of variables is available in the Appendix A.

Data were collected and managed using REDCap electronic data capture tools hosted at “Instituto de Estudio de Ciencias de la Salud de Castilla y León”, Spain [30]. The manuscript was adapted to the STROBE statement. This study was conducted in accordance with the ethical standards laid down in the 1964 Helsinki Declaration and received approval from the institutional review boards of the coordinating center and each participating hospital. This study is registered at ClinicalTrials.gov (NCT04663893).

Quantitative variables were summarized as medians and interquartile ranges (IQRs), and qualitative variables were presented according to their frequency distribution and percentages. Differences between groups were assessed using Pearson’s chi-square test or Fisher’s exact test for categorical variables, and the Mann–Whitney U test for continuous variables. Multivariate Cox regression analyses were performed to identify independent risk factors for 1-year mortality after VC-PFF. Odds ratios (ORs) and 95% confidence intervals (CIs) were reported. Collinearity tests were performed before looking for an adequate multivariate model. An ROC analysis was used for the multivariate model. The Kruskal–Wallis test was used to find differences in one-year follow-up EQ5D between management strategies. *p*-values < 0.05 were considered statistically significant. Statistical analyses were performed using SPSS v.29 software (IBM, Armonk, NY, USA).

## 3. Results

### 3.1. Patient Demographics and Preoperative Characteristics

A total of 262 patients with Vancouver type C periprosthetic femoral fractures were included. The median age was 85 years (interquartile range (IQR) 12.75), and 77.1% (*n* = 202) were female. Patients were generally frail, with a median Clinical Frailty Scale (CFS) score of 5 (IQR 2) and exhibited mild cognitive impairment (median Pfeiffer score 3, IQR 5). The median age-adjusted Charlson comorbidity index (a-CCI) was 6 (IQR 2), and the median admission hemoglobin level was 12.1 g/dL. Most patients (77.9%) were community dwellers prior to the fracture, and 63.9% had independent outdoor mobility (Table 1).

### 3.2. Surgical Management

Surgery was performed in 94.7% (*n* = 248) of cases. Among these, 62.3% underwent internal fixation with a locking plate, and 29.1% received a retrograde intramedullary nail. Prosthesis revision was required in only 4.4% of patients. Neuroaxial anaesthesia was the most common technique (70.7%), and 53.8% of procedures employed an open approach. Cerclage wires for reduction were used in 41.7% of operations (Table 2).

### 3.3. Acute Clinical Management and Post-Operative Care

Acute clinical management and post-operative care are shown in Table 3. Half of the patients had a geriatrician involved in their management, and 77.8% of the patients were moved out of bed within the first 48 h after the operation. However, only 36.2% of the patients were able to ambulate before discharge.

### 3.4. Postoperative Course and One-Year Outcomes

**Figure 1:**
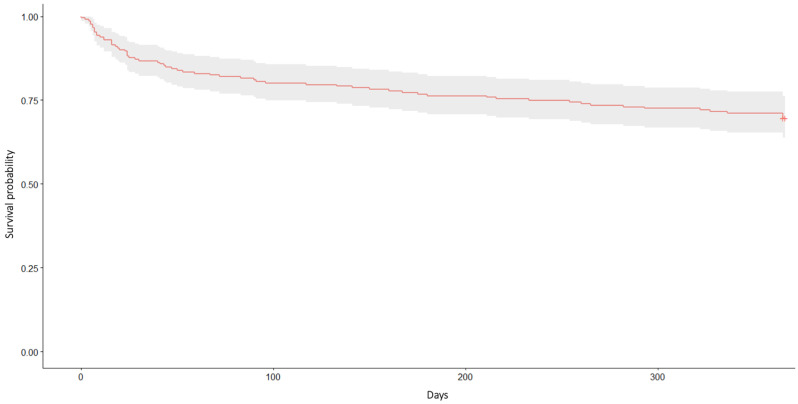
Survival probability during the first year post-operatively, illustrated by the Kaplan–Meier curve.

Cumulative mortality rates for patients with VC-PFFs were as follows: 7.3% (*n* = 19) in-hospital, 13.0% (*n* = 34) within 30 days, 19.7% (*n* = 51) within six months, and 28.4% (*n* = 74) within one year, as illustrated by the Kaplan–Meier curve in Figure 1.

At one year, 75.5% of survivors resided at home, and 54.3% had regained independent outdoor mobility. Fracture union was achieved in 83% of cases. Surgical complications were infrequent, with an overall rate of 11% at one year. Infection occurred in 4.5% of cases, and 4.5% of the patients underwent surgery for the treatment of non-union.

Medical complications occurred in 51.9% of patients during hospitalization, with delirium (20.6%), respiratory (16.0%), and renal (15.3%) issues being the most frequent. Early sitting (<48 h post-op) was achieved in 77.8% of patients. At discharge, 57% of patients returned home, while 43% were institutionalized. A total of 36% of the patients were discharged without antiosteoporotic treatment (Table 4).

The comparison of one-year outcomes between patients surgically treated and patients conservatively managed is shown in Table 5. The main differences between both treatment strategies were worse one-year mobility, more weight-bearing restrictions at one month, but fewer complications needing surgical treatment in patients conservatively managed.

Weight-bearing restrictions at hospital discharge and at 30-day follow-up were higher for patients treated with plates (*p* < 0.001 and *p* < 0.001) than for patients fixed with nails, but there were no differences in mobility decline at one-year follow-up between both fixation techniques (*p* = 0.776).

There were no differences in quality of life at one-year follow-up EQ5D between patients receiving surgical treatment and non-surgical treatment, between fixation and locked plate versus nail, and between patients who received co-management with geriatricians and those who did not (Table 6).

### 3.5. Predictors of Mortality and Complications

**Figure 2:**
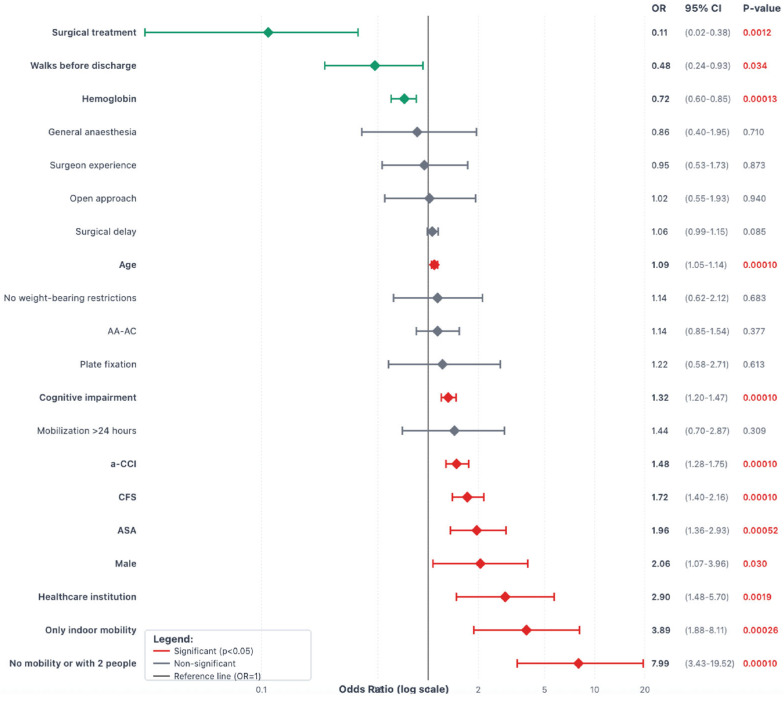
Forest plot diagram with predictors for one-year mortality in patients with Vancouver C-type periprosthetic femoral fractures. Interpretation: Variables to the left of the black vertical reference line (OR < 1) are protective factors. Variables to the right of the black reference line (OR > 1) are risk factors. Confidence intervals that do not cross the reference line are statistically significant. Green diamonds indicate significant protective factors; red diamonds indicate significant risk factors. Statistically significant *p*-values are highlighted in red.

Univariate logistic regression identified the following risk factors for one-year mortality: male sex (OR 2.06, *p* = 0.03), age (OR 1.08 per year, *p* < 0.001), CFS (OR 1.72, *p* < 0.001), living at a healthcare institution (OR 2.9, *p* = 0.002), only indoor mobility (OR 3.89, *p* < 0.001), no mobility or with the help of two people (OR 7.99, *p* < 0.001), cognitive impairment (Pfeiffer score OR 1.32, *p* < 0.001), a-CCI (OR 1.48, *p* < 0.001), and ASA score (OR 1.96, *p* < 0.001). Protective factors included higher hemoglobin at admission (OR 0.72, *p* < 0.001), surgical treatment (OR 0.11, *p* = 0.001), and ambulation before hospital discharge (OR 0.48, *p* = 0.034). The type of surgical fixation (plate vs. nail) did not show significant associations with mortality. Similarly, surgical approach, anesthesia type, or surgeon experience did not influence outcomes (Figure 2).

**Figure 3:**
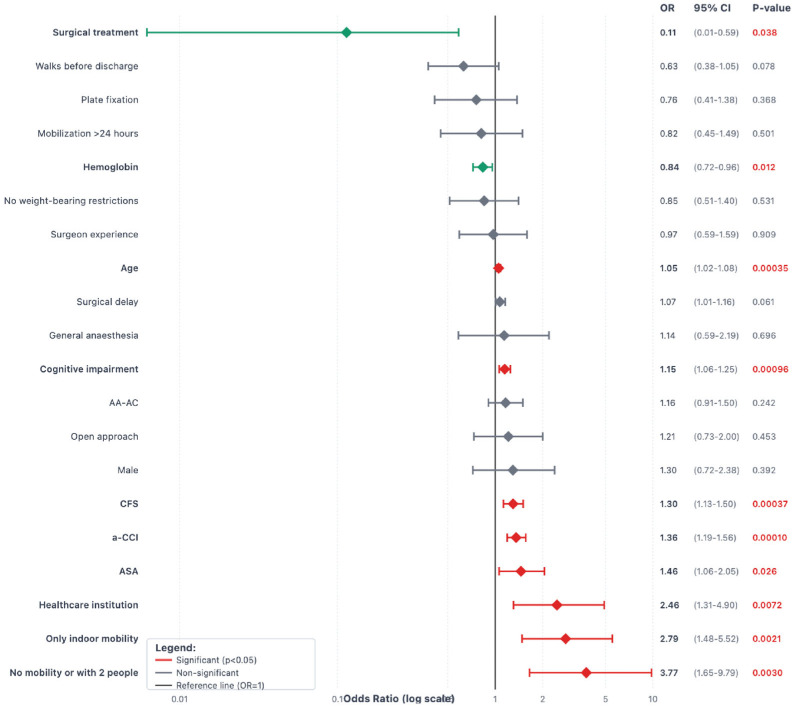
Forest plot diagram with predictors for medical complications during the first year after a Vancouver C-type periprosthetic femoral fracture. Interpretation: Variables to the left of the black vertical reference line (OR < 1) are protective factors. Variables to the right of the black reference line (OR > 1) are risk factors. Confidence intervals that do not cross the reference line are statistically significant. Green diamonds indicate significant protective factors; red diamonds indicate significant risk factors. Statistically significant *p*-values are highlighted in red.

**Figure 4:**
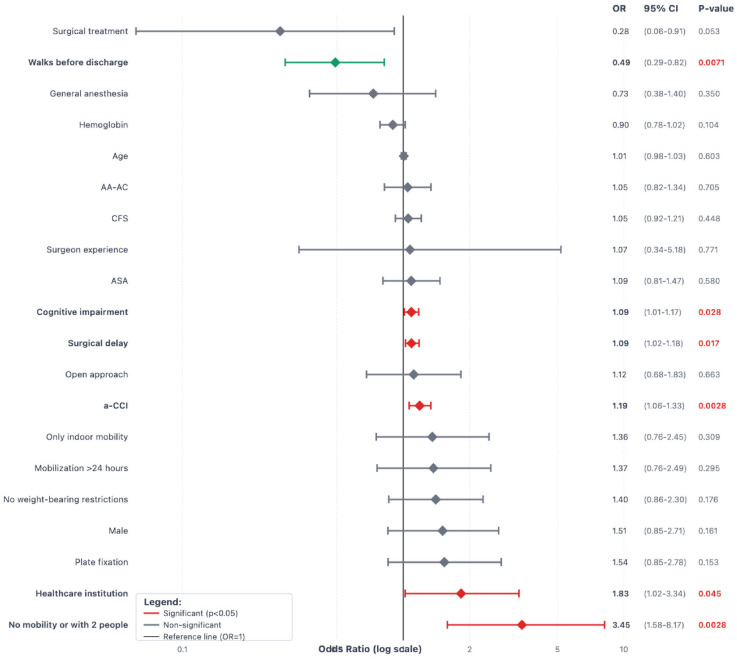
Forest plot diagram with predictors for surgical complications during the first year after a Vancouver C-type periprosthetic femoral fracture. Interpretation: Variables to the left of the black vertical reference line (OR < 1) are protective factors. Variables to the right of the black reference line (OR > 1) are risk factors. Confidence intervals that do not cross the reference line are statistically significant. Green diamonds indicate significant protective factors; red diamonds indicate significant risk factors. Statistically significant *p*-values are highlighted in red.

**Figure 5:**
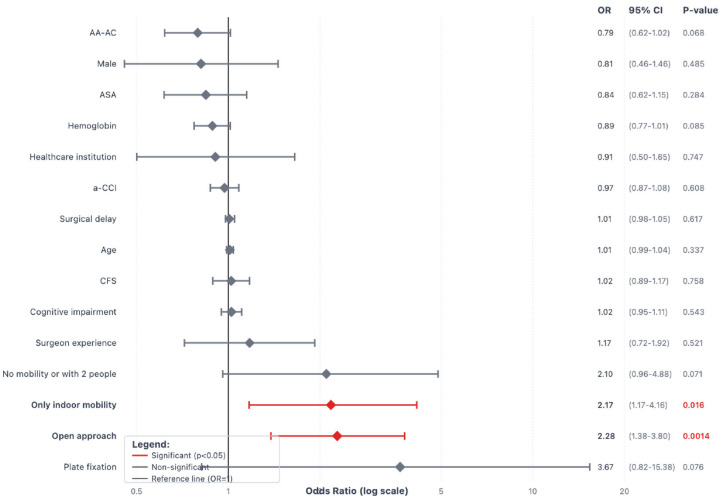
Forest plot diagram with predictors for weight-bearing restrictions in patients with Vancouver C-type periprosthetic femoral fractures. Interpretation: Variables to the left of the black vertical reference line (OR < 1) are protective factors. Variables to the right of the black reference line (OR > 1) are risk factors. Confidence intervals that do not cross the reference line are statistically significant. Green diamonds indicate significant protective factors; red diamonds indicate significant risk factors. Statistically significant *p*-values are highlighted in red.

Figure 3, Figure 4 and Figure 5 show the forest plot diagrams with predictors for medical complications and surgical complications during the first year and risk factors for weight-bearing restrictions. Fragility variables were all predictors for medical complications, while only cognitive impairment, a-CCI, and no mobility before the fracture were risk factors for surgical complications. An open approach was the only risk factor for weight-bearing restrictions (OR 2.28, *p* = 0.001).

The logistic regression analysis with the influence of a geriatrician in the management showed that not having a geriatrician involved in the management of VC-PFFs was a protective factor for the presence of medical complications, OR 0.564 (95% CI 0.343–0.924, *p* = 0.023). The risk was not significant for one-year mortality OR 0.614 (95% CI 0.336–1.108, *p* = 0.108), surgical complications OR 0.896 (95% CI 0.555–1.445, *p* = 0.651), and weight-bearing restrictions OR 0.758 (95% CI 0.463–1.238, *p* = 0.270). Table 7 shows the logistic regression analysis with the risk for 30-day and 1-year mortality in the presence of medical complications during hospital stay.

**Figure 6:**
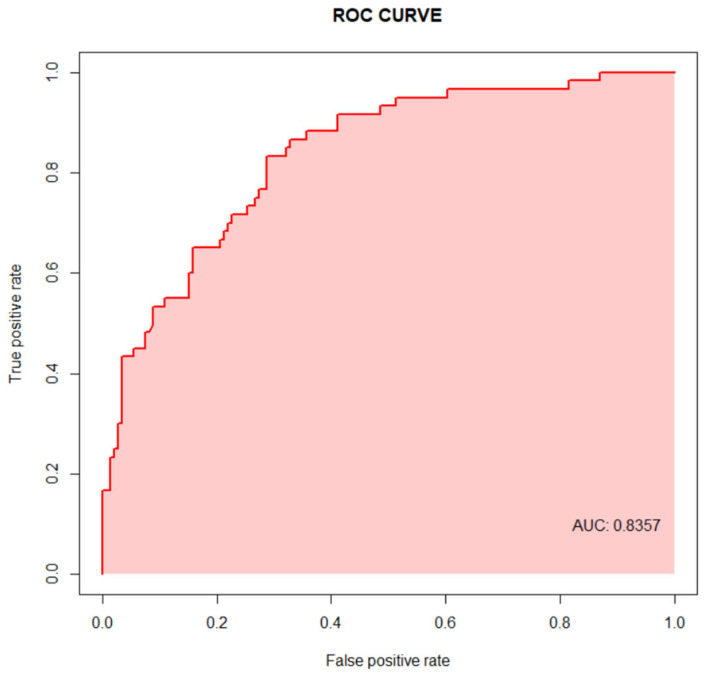
ROC curve for the one-year mortality multivariate model used. AUC, area under the curve.

Multivariate analysis showed that age, being male, cognitive impairment, ASA, and a-CCI were risk factors for mortality during the first year post-fracture (Table 8). Given the potential interdependence among CFS, Pfeiffer score, ASA, and a-CCI as predictors of one-year mortality, collinearity tests were performed before looking for an adequate multivariate model. Alternative modelling approaches were explored (Cox regression with hazard ratios and decision tree-based models such as CART or random forest), but failed to optimize the model’s predictive performance. The ROC analysis for the multivariate model, which was finally used, had an area under the curve (AUC) of 0.8356735, being good at discriminating between patients who died and those who survived (Figure 6).

## 4. Discussion

This prospective multicenter study provides one of the largest and most detailed analyses to date of VC-PFFs, a relatively underrepresented subset of periprosthetic fractures in the orthopedic literature [1,5,7,11,26,31,32,33,34,35,36,37,38]. Our findings confirm the high one-year mortality rate associated with these injuries and identify key demographic, clinical, and perioperative factors that significantly influence outcomes.

### 4.1. Mortality and Risk Factors

The observed one-year mortality rate of 28.4% aligns with or exceeds prior reports for VC PFFs and is comparable to outcomes seen in geriatric hip fractures. Previous studies have reported variable mortality rates, ranging from 15% to 27%, in similar cohorts, often due to differences in patient age, comorbidities, and study design [6,7,8,18,22,39,40]. Our cohort’s higher median age (85 years) and clinical frailty may explain the elevated mortality. In our study population, the risk of mortality increased by 8% (*p* < 0.001) each year; thus, mortality rates are higher in elderly populations. VC-PFFs are more frequent in women, but the risk of mortality for males was double. Khan et al. found a higher risk of mortality in type B PFF for male and older patients than for young women [2].

Multivariate analysis confirmed that increasing age, male sex, cognitive decline, frailty, and greater comorbidity burden (a-CCI and ASA) are significant predictors of mortality. These findings are consistent with prior literature on hip fracture populations [14,16,18] and underscore the importance of comprehensive geriatric assessment in the perioperative period. More than half of the patients in this study were managed by a geriatrician, which does not necessarily represent the standard practice in the country. We think there is a hospital selection bias in the PIPPAS study, as many hospitals joining are also involved in the Spanish hip fracture registry, which is run mainly by geriatricians. Higher hemoglobin level at admission was a protective factor for mortality for patients with VC-PFFs. This finding goes in line with findings for peri-implant fractures of the femur [41] and hip fractures [42], and mortality associated with postoperative anemia [43].

Early ambulation and preserved functional mobility were protective factors. Patients who were able to walk before discharge had significantly reduced odds of death at one year. This supports that resistant fixation constructs should be used to allow early mobilization protocols and highlights functional recovery as a prognostic indicator.

### 4.2. Surgical Management and Fixation Type

Contrary to some prior registry-based analyses, our study did not find a statistically significant difference in mortality between patients treated with locking plates and intramedullary nails [20,21,22,24]. Chatziagorou et al. found lower mortality rates in patients treated with locking plates than those treated with nails [22]. While biomechanical and consolidation outcomes may favor one technique over the other in certain contexts, our data suggest that patient-related factors, rather than the fixation method, are the primary drivers of survival. Some studies group Vancouver B1 PFF and VC-PFF together as the stem is not usually revised [6,7,24,25]. However, treatment for B1 types is technically more complicated; therefore, these two types of fractures should be individually studied.

Surgical approach, type of anesthesia, and surgeon experience were not associated with mortality, showing that patient factors outweigh procedural variables in this population. Open approaches were associated with higher rates of weight-bearing restrictions than less invasive approaches. Surgical treatment was a protective factor for better outcomes, but probably patients not fit for surgery were the frailer ones with more medical and surgical complications and therefore with less odds for survival.

### 4.3. Fracture Prevention Strategies

A total of 34.4% of the patients were under any kind of antiosteoporosis treatment when the VC-PFF occurred, and 64% received any treatment for osteoporosis at hospital discharge. This rate remained similar during the one-year follow-up, with a high compliance rate for this aged population. The secondary prevention of fragility fractures in the VC-PFF population showed a critical systemic gap that needs to be addressed. More than one-third of the patients were not receiving any treatment for osteoporosis at any time point, and only one out of four patients was under anabolic or antiresorptive medication at any time point. These findings support the integration of secondary fracture prevention strategies into postoperative care pathways, in line with the Fracture Liaison Service (FLS) model. Aggressive bone health optimization, including formal osteoporosis assessment, initiation of anti-osteoporosis therapy when indicated, and periodic reassessment of vitamin D and calcium, combined with multifactorial fall-prevention programs, can help reduce imminent refracture risk and facilitate rehabilitation.

### 4.4. Complications and Their Impact

Medical complications during the initial hospital stay and in the early postoperative period were strongly associated with increased mortality (*p* < 0.001). These included cardiac, respiratory, renal, and neurological issues, consistent with previous findings in the fragility fracture literature [3,6,12,24,27]. Similarly, early surgical complications, although less frequent, were linked to poor outcomes. The data gathered by the PIPPAS study shows that when there was a geriatrician involved in the management of the patient, both from the beginning in an orthogeriatric unit, or if the patient had medical complications and a geriatrician was involved afterwards. Therefore, we could not perform a specific analysis on the benefits of an orthogeriatric unit. The logistic regression analysis showed that when a geriatrician was not involved, there was a protective effect for medical complications, but probably in certain cases, the geriatrician was involved because there was already a medical complication. The presence of medical complications in the acute setting increased the risk of thirty-day and one-year mortality. This information is relevant for both preventive interventions and adequate management once the complication happens. The increased risk of mortality associated with medical complications highlights the importance of geriatric and orthopaedic co-management. As seen in previous reports [10,12], our results support that postoperative monitoring by a multidisciplinary team, including geriatricians, and management of medical complications in VC-PFF should be prioritized to improve survival.

### 4.5. Functional Outcomes and Discharge Planning

Most patients who survived one year after VC PFF had regained independent outdoor mobility and returned home. Institutionalization—both pre-fracture and at discharge—was a significant predictor of mortality. This supports the role of social determinants and functional status in long-term outcomes and highlights the importance of discharge planning and community support. Langenhan et al. found weight-bearing restrictions related to mortality [40], but we did not find this association. Previous analysis from the PIPPAS study group on all lower limb periprosthetic fractures showed an association between weight-bearing restrictions and smaller odds for returning to the community. From a biomechanical perspective, the comparison between locked plating and retrograde nailing was reported as not significantly associated with mortality. However, patients fixed with locked plates showed higher rates of weight-bearing restrictions than patients fixed with nails, limiting early mobilization and its advantages. However, this fact might not have influenced the final functional recovery, with no differences in mobility decline and EQ5D at one-year follow-up between both fixation techniques. Nevertheless, it remains unclear what the real influence of these techniques on early mobilization and functional recovery is when weight-bearing restrictions are established by the surgeon, mainly based on subjective impressions. We could not demonstrate differences in quality of life (EQ5D) at one-year follow-up between the different management strategies, surgical treatment, type of fixation, and geriatric co-management.

The association between mobility and fracture healing was not explicitly assessed in this study, but it may warrant future investigation.

### 4.6. Strengths and Limitations

A key strength of this study is its prospective, multicenter design and large sample size, which enhances generalizability. The use of standardized data collection across 59 centers and rigorous statistical analysis further strengthens the validity of the findings. Nonetheless, to the best of our knowledge, and excluding meta-analyses, this represents the largest series of VC-PFF cases reported to date.

However, several limitations must be acknowledged. First, despite prospective data capture, some variables were subject to reporting bias or local clinical interpretation. Second, there was variability in the treatment and management methods that were employed. Third, the decision on the fixation method used was made by the attending surgeon based on the patient’s functional status, comorbidities, and the fracture pattern. Finally, while the study focused on mortality and complications, quality-of-life outcomes were not assessed and should be considered in future research.

## 5. Conclusions

In conclusion, VC-PFFs were associated with significant one-year mortality. Advanced age, male sex, frailty, cognitive impairment, and high comorbidity burden were independent risk factors for mortality. Early postoperative ambulation and high hemoglobin level at admission reduced the risk of mortality.

The type of internal fixation was not associated with the outcomes, but minimally invasive surgical approaches showed less weight-bearing restrictions.

Medical and surgical complications were powerful predictors of mortality and should be a focus of postoperative surveillance and intervention.

Taken together, these results provide a foundation for a stratified and patient-centered approach to the management of VC-PFFs. Future prospective studies with longer-term follow-up are warranted to refine treatment algorithms and improve outcomes in this increasingly prevalent clinical scenario.

## Figures and Tables

**Table 1:** Demographic and baseline data for patients presenting with a Vancouver type C periprosthetic femoral fracture. Qualitative variables are summarized using counts and percentages (%). Continuous variables were summarized as median and interquartile range (IQR). Categorical variables were summarized by absolute frequencies and percentages.

Age (years) Median—IQR	85	12.75
Gender—no. %		
Female	202	77.1
Male	60	22.9
Place of residency—no. %		
Comunity dweller	204	77.9
Healthcare institution	58	22.2
Pfeiffer’s SPMSQ Median—IQR	3	5
CFS Median—IQR	5	2
a-CCI Median—IQR	6	2
ASA—no. %		
1	5	1.9
2	58	22.1
3	156	59.5
4	36	13.7
5	0	0
Osteoprotective treatment—no. %		
No treatment	172	65.6
Anti-resorptive	12	4.6
Bone-forming	6	2.3
Calcium	55	21
Vitamin D	76	29
Antiaggregant or anticoagulant medication ^—no. %		
None	164	62.8
Acenocumarol or NOAC or PAA	95	36.4
Double	2	0.8
Hb at admission (gr/dL) Median—IQR	12.1	2.4
Pre-Fracture mobility—no. %		
Independent gait oudoors (w/wo aids)	167	63.9
Independent gait indoors	56	21.4
No mobility or only with help of two people	36	13.8
Cemented stem—no. %		
Cemented	150	57.5
Uncemented	111	42.5

IQR interquartile range, Pfeiffer′s SPMSQ Pfeiffer′s Short Portable Mental Status Questionnaire, CFS clinical frailty scale, age-adjusted Charlson comorbidity Index, ASA American Society of Anesthesiologists (ASA) physical status classification system, NOAC new oral anti-coagulant, PAA platelet anti-aggregant, Hb hemoglobin. ^ Osteoprotective treatment: the percentage for each individual treatment was calculated with respect to the total number of patients receiving osteoprotective treatment.

**Table 2:** Management of Vancouver type C periprosthetic femoral fractures. Categorical variables are summarized as absolute frequencies and percentages from the number of patients surgically managed in each group, except for “treatment”. Qualitative variables are summarized using counts and percentages (%). Continuous variables were summarized as median and interquartile range (IQR).

Treatment	n	%
Operative	248	94.7
Non-operative	14	5.3
Type of anaesthesia		
General	44	17.9
Spinal	174	70.7
Regional	48	19.5
Surgical approach		
Open	133	53.8
MIS	53	21.5
PC	62	24.7
Cerclage for reduction		
Yes	103	41.7
No	144	58.3
Stem revision		
Yes (cementless)	6	2.4
Yes (cemented)	5	2.0
No	236	95.5
Type of Fixation		
1 Plate	148	59.9
2 Plates	6	2.4
Nail	72	29.1
Cerclage	45	18.2
Isolated Screws	3	1.2
Surgeon experience		
>20 Replacements	59	24.0
>20 MIPO	80	32.5
<20 Replacements and MIPO	115	46.7

IQR, interquartile range; MIS, minimally invasive surgery; PC, percutaneous; MIPO, minimally invasive plating osteosynthesis.

**Table 3:** Acute clinical management and post-operative care data. Categorical variables are summarized as absolute frequency and percentages from the number of patients in each category. Qualitative variables are summarized using counts and percentages (%). Continuous variables were summarized as median and interquartile range (IQR). IQR: interquartile range.

Medical Staff Involved in the Patient Care (Other Than Trauma and Anaesthesia) no. %		
Geriatrician	106	40.5
Internal Medicine	73	27.9
Geriatrician and others	31	11.8
Others	15	5.7
None	37	14.1
Initial postoperative mobilization out of bed—no. %		
<48 h	196	77.8
>48 h	56	22.2
Ability to walk at hospital discharge—no. %		
Yes	92	36.2
No	158	62.2
Post-op hemoglobin Median. IQR	9.5	1.8
Management of the anaemia—no. %		
No	62	23.8
Transfusion	171	65.8
Intravenous iron	73	28.1

**Table 4:** Mortality and outcomes for all patients, including patients non-surgically treated, during hospital stay, at hospital discharge, at 30 days, 6 months, and 1-year follow-up (fu). Qualitative variables are summarized using counts and percentages (%). Continuous variables were summarized as median and interquartile range (IQR). Categorical variables were summarized by absolute frequencies and percentages. IQR, interquartile range. N/A not available. + The percentages for the different osteoprotective treatments at discharge referred to the total number of patients who were receiving treatment.

Variable	Hospital	30 Days fu	6 Months fu	1-y fu
Mortality—no. (%)	19 (7.3)	15 (6.7)	17 (8.7)	23 (5.7)
CFS Median. (IQR)	-	-	6 (3)	6 (3)
Pfeiffer Median (IQR)	-	-	-	6 (2)
Mobility—no (%)				
Independent gait outdoors (w/wo aids)		53 (24.1)	92 (51.4)	77 (54.3)
Independent gait indoors		51 (23.1)	40 (22.3)	28 (19.7)
No mobility or only with help of two people		113 (51.1)	47 (26.3)	36 (25.4)
Medical complications—no (%)				
No	126 (48.1)	204 (87.9)	160 (82.5)	122 (78.7)
Yes (any)	136 (51.9)	28 (12.1)	34 (17.5)	33 (21.3)
Cardiac	29 (11.1)	3 (1.3)	12 (6.2)	13 (8.4)
Respiratory	42 (16.0)	7 (3.0)	10 (5.2)	10 (6.5)
Pulmonary thromboembolism	1 (0.4)	0 (0)	1 (0.5)	0 (0)
Renal	40 (15.3)	5 (2.2)	5 (2.6)	5 (3.2)
Neurological	5 (1.9)	4 (1.7)	3 (1.5)	4 (2.6)
Gastrointestinal	31 (11.8)	5 (2.2)	6 (3.1)	2 (1.3)
Urinary tract infection	34 (13.0)	10 (4.3)	8 (4.1)	6 (3.9)
Delirium	54 (20.6)	7 (3.0)	4 (2.1)	7 (4.5)
Surgical complications—no (%)				
No		221 (95.3)	175 (91.1)	138 (89.0)
Fracture		1 (0.4)	2 (1.0)	1 (0.6)
Dislocation		0 (0)	0 (0)	0 (0)
Infection		9 (3.9)	4 (2.1)	7 (4.5)
Failure of fixation		1 (0.4)	7 (3.6)	5 (3.2)
Loosen prosthesis		0 (0)	0 (0)	1 (0.6)
Nonunión		N/A	7 (3.6)	7 (4.5)
Weight-bearing restrictions—no. %				
No restrictions	109 (42.9)	117 (52.9)	-	-
Only tranferences or not allowed	145 (57.1)	104 (47.1)	-	-
Place of residence—no (%)				
Community dweller	138 (57.0)	136 (61.8)	121 (66.5)	108 (75.5)
Healthcare institution	104 (43.0)	83 (37.7)	60 (32.9)	35 (24.5)
Osteoprotective treatment +—no. %				
No treatment	87 (36.0)	82 (37.3)	64 (35.2)	53 (37.1)
Anti-resorptive	54 (22.3)	46 (20.9)	38 (20.9)	29 (20.3)
Bone-forming	12 (5.0)	15 (6.8)	11 (6)	5 (3.5)
Calcium	105 (43.4)	99 (45)	82 (45.1)	63 (44.1)
Vitamin D	138 (57.0)	123 (55.9)	106 (58.2)	81 (56.6)

**Table 5:** Comparison of one-year outcomes between patients surgically treated and patients conservatively managed. Qualitative variables are summarized using counts and percentages (%). Continuous variables were summarized as median and interquartile range (IQR). Categorical variables were summarized by absolute frequencies and percentages. IQR, interquartile range. + The percentages for the different osteoprotective treatments at discharge referred to the total number of patients who were receiving treatment. N/A not applicable, for a low number of cases.

Variable	Surgical	Conservative	*p*
Mortality—no. (%)	15 (9.38)	0 (0)	N/A
CFS Median. (IQR)	6(3)	6 (2.5)	0.9987
Pfeiffer Median (IQR)	2(3)	3 (3.5)	0.1287
Mobility—no (%)			
Independent gait oudoors (w/wo aids)	169 (66.27)	3 (21.43)	<0.001
Independent gait indoors	57 (22.35)	4 (28.57)	<0.001
No mobility or only with help of two people	29 (11.37)	7 (50)	<0.001
Medical complications—no (%)			
No	124 (48.44)	13 (92.86)	0.006
Yes (any)	132 (51.56)	1 (7.14)	0.006
Cardiac	13 (5.08)	0	N/A
Respiratory	9 (3.52)	1 (7.14)	1
Pulmonary thromboembolism	0	0	N/A
Renal	5 (1.95)	0	N/A
Neurological	4 (1.56)	0	N/A
Gastrointestinal	2 (0.78)	0	N/A
Urinary tract infection	4 (1.56)	2 (14.29)	0.027
Surgical complications—no (%)			
No	139 (54.3)	0 (0)	N/A
Fracture	1 (0.39)	0 (0)	N/A
Dislocation	0	0 (0)	N/A
Infection	7 (2.73)	0 (0)	N/A
Failure of fixation	5 (1.95)	0 (0)	N/A
Loosen prosthesis	1 (0.39)	0 (0)	N/A
Nonunión	7 (2.73)	0 (0)	N/A
Weight-bearing restrictions 30 days			
No restrictions	112 (43.92)	0	-
Only transferences or not allowed	143 (56.08)	14 (100)	0.048
Place of residence			
Community dweller	107 (74.83)	2 (66.67)	1
Healthcare institution	36 (25.17)	1 (33.33)	1
Osteoprotective treatment +			
No treatment	53 (20.7)	2 (14.29)	1
Anti-resorptive	29 (11.33)	2 (14.29)	1
Bone-forming	6 (2.34)	0	N/A
Calcium	64 (25)	2 (14.29)	1
Vitamin D	81 (31.64)	3 (21.43)	1

**Table 6:** One-year follow-up EQ5D differences between management strategies. Median and interquartile range.

Surgical Treatment 0.6 (0.39)	vs.	Non-Operative 0.25 (0.45)	*p*-Value 0.457
Locked Plate 0.59 (0.47)	vs.	Nail 0.59 (0.31)	0.904
Geriatrician 0.59 (0.44)	vs.	No co-management 0.61 (0.4)	0.385

**Table 7:** Risk for 30-day and one-year mortality associated with the presence of medical complications during the acute setting. OR, odds ratio; CI, confidence Interval.

	Risk of 30-Day Mortality			Risk of 1-Year Mortality		
Medical Complication	OR	CI	*p*-Value	OR	CI	*p*-Value
Cardiac	6.698	(0.993, 3.917)	<0.001	1.981	(0.993, 3.917)	0.049
Respiratory	2.309	(0.944, 5.281)	0.054	4.030	(1.913, 8.673)	<0.001
Renal	3.448	(1.477, 7.750)	0.056	2.078	(0.969, 4.407)	0.056
Neurological	31.448	(4.468, 626.6)	0.002	9.800	(1.414, 193.8)	0.043
Gastrointestinal	1.394	(0.444, 3.670)	0.530	1.101	(0.428, 2.629)	0.834
Urinary tract infection	3.548	(1.460, 8.181)	0.004	3.179	(1.412, 7.254)	0.005
Delirium	2.812	(1.275, 6.051)	0.008	1.981	(0.993, 3.917)	0.049

**Table 8:** Cox’s regression multivariate analysis for one-year mortality.

Variable	OR	CI (95%)	*p*-Value
Age	1.05	(1.00, 1.11)	0.057
Male	2.25	(0.97, 5.25)	0.058
Cognitive impairment (Pfeiffer’s SPMSQ)	1.27	(1.14, 1.43)	<0.001
ASA	1.61	(1.01, 2.55)	0.042
a-CCI	1.32	(1.11, 1.59)	0.002

OR odds ratio, CI confidence interval, Pfeiffer′s SPMSQ Pfeiffer′s Short Portable Mental Status Questionnaire, ASA American Society of Anesthesiologists (ASA) physical status classification system, a-CCI age-adjusted Charlson comorbidity index.

## Data Availability

The raw data supporting the conclusions of this article will be made available by the authors on request.

## Abbreviations

The following abbreviations are used in this manuscript:

PFF	Periprosthetic Femoral Fracture
VC-PFF	Vancouver type-C Periprosthetic Femoral Fracture
UCS	Unified Classification System
PIPPAS	Peri-Implant and PeriProsthetic Fracture Analysis for Survival
SPMSQ	Short Portable Mental Status Questionnaire
IQR	Interquartile ranges
CFS	Clinical Frailty Scale
a-CCI	age-adjusted Charlson comorbidity index
ASA	American Society of Anesthesiologists’ physical status classification system
NOAC	new oral anti-coagulant
PAA	platelet anti-aggregant,
Hb	hemoglobin
MIS	Minimally Invasive Surgery
PC	Percutaneous
MIPO	Minimally Invasive Plating Osteosynthesis
OR	Odds Ratio

## Appendix A

Comprehensive list of data collected from patients presenting with a Periprosthetic fracture

BLOCK 1. Epidemiology, Health status, and in-hospital management variables:

1.1 Date of birth

1.2 Gender

1.3 Pre-fracture place of residence: own home, nursing facility, acute hospital, N/A

1.4 Pre-fracture mobility: (FFN-MCD scale)

1 completely independent gait

2 outdoors independent gait with 1 technical aid

3 outdoors independent gait with 2 technical aids

4 only indoors independent gait w or w/o aids

5 no mobility at all or with the help of 2 other people

6 N/A

1.5 Mental assessment: Pfeiffer’s SPMSQ, Pfeiffer’s Short Portable Mental Status Questionnaire. Number of mistakes

1.6 Clinical Frailty Scale (CFS) (2 weeks pre-fracture).

1.7 ASA: I, II, III, IV, V, N/A

1.8 Charlson comorbidity index (CCI): Individualized organ/system punctuations are registered

1.9 Type of fracture: Periprosthetic, Peri-implant

If the bone hosts one prosthesis and one fixation device, choose the type of fracture that has the most influence on the treatment.

1.10 Are there more implants in the same bone? No, Prosthesis, Nail, Plate, Isolated screw.

1.11 Total number of fractures supported in the injured bone (including the actual fracture)?

1.12 Osteoprotective treatment: Anti-resorptive, Bone-forming, Calcium, Vitamin D, none.

1.13 Antiaggregant or anticoagulant medication:

1 NO,

2 Acenocumarol, NOAC, PAA (Clopidogrel/Ticlo/AAS 300) NOAC: New Oral Anti-Coagulant, PAA: Platelet Anti-Aggregant

3 Double

1.14 Date of Fracture

1.15 Date and time the patient is admitted to the emergency

1.16 Does the patient receive surgical treatment? Yes or No

Date and time of surgical treatment

1.17 Haemoglobin level (g/dL): At admission and 1st day post-op

1.18 Medical Complications during hospital stay: (which may need treatment)

Cardiac, respiratory, Pulmonary thromboembolism, Urinary infection, Renal, Brain, delirium, Gastrointestinal, in-hospital fractures, None

(Multiple answers are possible except none)

1.19 Co-management with other specialties: (apart from traumatology and anaesthesia): Geriatrics, Internal Medicine, other specialties, Geriatrics and Other specialties, None

1.20 Did the patient sit down during the first day post-op? If the patient was managed non-surgically, did the patient sit the day after the decision? Yes or No

1.21 Was full weight bearing allowed?

No restrictions or with external aids in elderly patients

Only for transferences

Complete restriction (wheelchair in elderly patients or two crutches in young patients)

1.22 Was the patient walking at hospital discharge? Yes or No (either with or without weight-bearing restrictions)

1.23 In-hospital Mortality: Alive, dies before surgical treatment, dies in the operating room, dies post-operatively

1.24 Hospital discharge: Date and Time

1.25 Destination at hospital discharge:

Own home

Healthcare institution

Acute hospital

N/A

1.26 Osteoprotective treatment at hospital discharge: Anti-resorptive, Bone-forming, Calcium, Vitamin D, none. (Multiple answers are possible except none)

BLOCK 2. DIAGNOSIS Variables

2A Periprosthetic fractures diagnosis

2A.1 USP Classification:

2A.1.1 Bone:

Humerus Scapula       Forearm       Pelvis       Femur           Tibia         Patella

2A.1.2 Joint:

Shoulder       Elbow       Hip       Knee       Ankle

2A.1.3 Type:

A1         A2       B1     B2     B3         C       D     F

2A.2 Date when the prosthesis was implanted

2A.3 Previous Infection? Yes or No

2A.4 Was the prosthesis loose previously? Yes or No

2A.5 Is the prosthesis cemented? Yes or No

2A.6 Was the prosthesis painful previously? Yes or No

2A.7 Were there radiological signs of loosening previously? Yes or No

2A.8 Does the prosthesis have a stem? Yes or No

2A.9 Does the bone host a hip arthroplasty? No         Yes, stem prosthesis         Yes, stemless prosthesis         

2A.10 Does the bone host a knee arthroplasty? No         Yes, stem prosthesis         Yes, stemless prosthesis

BLOCK 3. TREATMENT Variables

3A PeriProsthetic Fracture Treatment

3A.1 Approach: Percutaneous       MIS-Hypo-invasive         Open

(Percutaneous: as for a small incision for a nail; Hypo-invasive: several incisions of the minimum size needed)

3A.2 Was the stability/fixation of the prosthesis checked?

Yes, from the joint                   Yes, from the fracture site                   No

3A.3 Was a cerclage used as a reduction tool? Yes or No

3A.4 Was revision of the prosthesis the treatment option? No           Yes, cementless                   Yes, cemented

3A.5 Was fixation the treatment option? No           Yes, 1 Plate       Yes, 2 Plates     Yes, Nail

Yes, definitive external fixator         Yes, cerclage         Yes, isolated screws      (Multiple answers option)

3A.6 Is there any overlap between implants? And length in millimeters

Overlap +         Kissing 0         Gap –               ___ mm

3A.7 Was a bone graft used? No           Yes, Strut             Yes, cancellous/reaming product

3A.8 Surgeon experience? >20 arthroplasty revisions in the last 12 months           >20 MIPO surgeries in the last 12 months               None of the previous (multiple answers are possible except none)

3A.9 Anaesthesia? General Neuro-axial         Regional       Different from previous

BLOCK 4. 30 DAYS FOLLOW-UP (from surgical treatment or from diagnosis if non-surgical treatment)

4A.1 Alive at 30-day follow-up? Yes or no

4A.1.2 Date of death

4A.2 Is weight bearing allowed?

No restrictions or with external aids in the elderly

Only for transferences

Weight bearing is forbidden (wheelchair in the elderly or crutches in young patients)

4A.3 Mobility at 30-day follow-up: (FFN-MCD scale)

1 completely independent gait

2 outdoors independent gait with 1 technical aid

3 outdoors independent gait with 2 technical aids

4 only indoors independent gait w or w/o aids

5 no mobility at all or with the help of 2 other people

6 N/A

4A.4 Any medical complication needing hospital admission within 30 days post-op?

☐NO         ☐Heart         ☐Respiratory         ☐Pulmonary thromboembolism             ☐Renal         ☐Cerebral     ☐Gastro-intestinal         ☐Any other

(Multiple answers are possible except none)

4A.5 Surgical complications at 30-day follow-up:

☐NO         ☐Fracture in the same bone     ☐Fixation failure       ☐Dislocation         

☐Loosen prosthesis           ☐Infection (Multiple answers are possible except none)

4A.6 Place of residence at 30-day follow-up: own home, nursing facility, acute hospital, N/A

4A.7 Osteoprotective treatment at 30-day follow-up: Anti-resorptive, Bone-forming, Calcium, Vitamin D, none. (Multiple answers are possible except none)

BLOCK 5. 6 MONTHS FOLLOW-UP (from surgical treatment or from diagnosis if non-surgical treatment)

5A.1 Alive at 6 months follow-up? Yes or no

5A.1.2 Date of death

5A.3 Mobility at 6 months follow-up: (FFN-MCD scale)

1 completely independent gait

2 outdoors independent gait with 1 technical aid

3 outdoors independent gait with 2 technical aids

4 only indoors independent gait w or w/o aids

5 no mobility at all or with the help of 2 other people

6 N/A

5A.4 Clinical Frailty Scale (CFS)

5A. Any medical complication needing hospital admission within 6 months post-op?

☐NO           ☐Heart         ☐Respiratory         ☐Pulmonary thromboembolism               ☐Renal         ☐Cerebral     ☐Gastro-intestinal         ☐Any other

(Multiple answers are possible except none)

5A.6 Surgical complications at 30-day follow-up:

☐NO           ☐Fracture in the same bone     ☐Fixation failure       ☐Dislocation         

☐Loosen prosthesis         ☐Infection (Multiple answers are possible except none)

5A.7 Is the fracture healed? Yes     No     Non-applicable (treated with a prosthesis)

5A.8 Place of residence at 6 months follow-up: own home, nursing facility, acute hospital, N/A

5A.9 Osteoprotective treatment at 30-day follow-up: Anti-resorptive, Bone-forming, Calcium, Vitamin D, none. (Multiple answers are possible except none)

BLOCK 6. 12 MONTHS FOLLOW-UP (from surgical treatment or from diagnosis if non-surgical treatment)

6A.1 Alive at 12 months follow-up? Yes or no

6A.1.2 Date of death

6A.3 Mental assessment: Pfeiffer’s SPMSQ, Pfeiffer’s Short Portable Mental Status Questionnaire. Number of mistakes

6A.4 Mobility at 12 months follow-up: (FFN-MCD scale)

1 completely independent gait

2 outdoors independent gait with 1 technical aid

3 outdoors independent gait with 2 technical aids

4 only indoors independent gait w or w/o aids

5 no mobility at all or with the help of 2 other people

6 N/A

6A.5 Clinical Frailty Scale (CFS)

6A.6 Any medical complication needing hospital admission within 6 months post-op?

☐NO           ☐Heart         ☐Respiratory         ☐Pulmonary thromboembolism             ☐Renal         ☐Cerebral     ☐Gastro-intestinal         ☐Any other

(Multiple answers are possible except none)

6A.7 Surgical complications at 30-day follow-up:

☐NO           ☐Fracture in the same bone     ☐Fixation failure     ☐Dislocation

☐Loosen prosthesis           ☐Infection (Multiple answers are possible except none)

6A.8 Is the fracture healed? Yes     No     Non-applicable (treated with a prosthesis)

6A.9 Place of residence at 12 months follow-up: own home, nursing facility, acute hospital, N/A

6A.10 Osteoprotective treatment at 12 months follow-up: Anti-resorptive, Bone-forming, Calcium, Vitamin D, none. (Multiple answers are possible except none)

6A.11 EQ5D

N/A: Non-Available, CFS: clinical frailty scale, ASA: American Society of Anesthesiologists (ASA) physical status classification system, NOAC: New Oral Anti-Coagulant, PAA: Platelet Anti-Aggregant, Hb: Haemoglobin.
